# Metabolomic profiling of patients with high gradient aortic stenosis undergoing transcatheter aortic valve replacement

**DOI:** 10.1007/s00392-020-01754-2

**Published:** 2020-10-14

**Authors:** Daniela Haase, Laura Bäz, Tarek Bekfani, Sophie Neugebauer, Michael Kiehntopf, Sven Möbius-Winkler, Marcus Franz, P. Christian Schulze

**Affiliations:** 1 Division of Cardiology, Angiology, Pneumology and Intensive Medical Care, Department of Internal Medicine I, University Hospital Jena, Friedrich-Schiller-University, Jena, Germany; 2 Department of Clinical Chemistry and Laboratory Diagnostics, University Hospital Jena, Friedrich-Schiller-University, Jena, Germany

**Keywords:** Metabolomics, Aortic stenosis, Transcatheter valve replacement, Prognostic marker

## Abstract

**Aim:**

Aim of our study was to evaluate metabolic changes in patients with aortic stenosis (AS) before and after transcatheter aortic valve replacement (TAVR) and to assess whether this procedure reverses metabolomic alterations.

**Methods:**

188 plasma metabolites of 30 patients with severe high-gradient aortic valve stenosis (pre-TAVR and 6 weeks post-TAVR) as well as 20 healthy controls (HC) were quantified by liquid chromatography tandem mass spectrometry. Significantly altered metabolites were then correlated to an extensive patient database of clinical parameters at the time of measurement.

**Results:**

Out of the determined metabolites, 26.6% (*n* = 50) were significantly altered in patients with AS pre-TAVR compared to HC. In detail, 5/40 acylcarnitines as well as 10/42 amino acids and biogenic amines were mainly increased in AS, whereas 29/90 glycerophospholipids and 6/15 sphingomyelins were mainly reduced. In the post-TAVR group, 10.1% (*n* = 19) of metabolites showed significant differences when compared to pre-TAVR. Moreover, we found nine metabolites revealing reversible concentration levels. Correlation with clinically important parameters revealed strong correlations between sphingomyelins and cholesterol (*r* = 0.847), acylcarnitines and brain natriuretic peptide (*r* = 0.664) and showed correlation of acylcarnitine with an improvement of left ventricular (LV) ejection fraction (*r* = − 0.513) and phosphatidylcholines with an improvement of LV mass (*r* = − 0.637).

**Conclusion:**

Metabolic profiling identified significant and reversible changes in circulating metabolites of patients with AS. The correlation of circulating metabolites with clinical parameters supports the use of these data to identify novel diagnostic as well as prognostic markers for disease screening, pathophysiological studies as well as patient surveillance.

**Electronic supplementary material:**

The online version of this article (10.1007/s00392-020-01754-2) contains supplementary material, which is available to authorized users.

## Introduction

Aortic stenosis (AS) is the most frequently occurring acquired heart valve disease in the western world showing increased prevalence rates with rising subject age. Over the last decade, the use of transcatheter aortic valve replacement (TAVR) has expanded and now represents the therapy of choice in elderly patients with symptomatic severe AS not only at high or moderate but also at low surgical risk [1–5]. Compared to surgical aortic valve replacement (SAVR), TAVR has benefits associated with shorter hospitalization, faster recovery and fewer re-hospitalization rates in patients with severe AS at a low or intermediate surgical risk and in younger (< 70 years) people, respectively [6–12]. Overall, the 30-day mortality after TAVR is 8.4% and the survival at 1- and 5-year amounts to 83% and 48%, respectively [13, 14]. Simultaneously, the development of new transcatheter valve platforms has improved procedural safety [15–17]. In conclusion, the indications for TAVR are expanding across the entire spectrum of operative risk, although data on long-term follow-ups of these implanted valves are still lacking [18].

While AS is a well-established clinical entity, detailed pathogenetic aspects still remain unclear. However, prediction of cardiac symptoms and adverse outcomes is complex in this elderly patient population suffering from a variety of comorbidities. Therefore, a promising additional approach is the investigation of the clinical impact of biomarkers to predict the efficiency of valve replacement and early intervention [19–21].

Profiling of circulating metabolites provides detailed insights into the metabolomic state of individual patients suffering from cardiovascular diseases. It can possibly contribute to (1) a more precise diagnosis, (2) a better prediction of individual prognosis, (3) therapeutical decision-making and (4) surveillance of therapy response [22–24]. When applied to well-diagnosed cohorts, these analytical techniques have the potential to elucidate the progression of metabolic perturbations in the heart. Recent studies on metabolomics of heart failure cohorts have shown alterations of metabolites, e.g. in dilated cardiomyopathy and coronary artery disease [25, 26]. In AS, altered metabolite profiles could be associated with acute kidney injury [27] and calcific aortic valve stenosis [28]. Systematic metabolite screening allows the characterization of new pathways for the discovery of diagnostic and prognostic markers or therapeutic targets in AS.

To address this challenge, we performed a targeted metabolomics approach to identify differences between high-risk AS patients at baseline and 6-week follow-up and in controls. We used high-throughput analysis to evaluate a panel of metabolites which were not yet investigated in this number and composition, particularly for glycerophospholipids and sphingomyelins. In this study, we give the first overview of metabolomics changes occurring in high-gradient aortic stenosis (HGAS) and within six weeks after TAVR, identifying reversible metabolic markers and correlations with an improvement of clinical parameters, thus identifying novel metabolite biomarkers that could be predictive of the clinical course.

## Materials and methods

### Patient population

A total of 30 consecutive patients with HGAS undergoing TAVR at the University Hospital Jena were included in this study. 20 patients without cardiovascular disease or other diseases except arterial hypertension and diabetes mellitus were included as healthy controls (HC). The diagnostic work-up of aortic stenosis was performed according to the current guidelines of the European Society of Cardiology (ESC) [2]. Transthoracic echocardiography was assessed in all patients prior to TAVR. Moreover, left ventricular ejection fraction (LVEF), LV end-diastolic diameter (LVEDD), LV end-systolic dimension (LVDS), interventricular septal thickness at end-diastole (IVSD), LV posterior wall thickness at end-diastole (LVPWD), LV mass and LV mass index (LVMI) were determined with baseline characteristics and laboratory parameters.

In the control group, malignant diseases, inflammatory, autoimmune or fibrotic disorders as well as status post pulmonary artery embolism, hyperthyroidism or intake of corticosteroids/immunosuppressive drugs were excluded.

Venous blood samples were obtained immediately before TAVR (pre-TAVR group) and 6-week follow-up (post-TAVR group). The samples were processed within 30 min after collection and the extracted EDTA plasma was stored in aliquots at − 80° until mass spectrometric measurements were performed. The study complies with the Declaration of Helsinki and the protocol was approved by the local ethics committee at the University Hospital Jena. All patients provided written informed consent (Registration Number: 4815-06/16).

### Laboratory and mass spectrometric analysis

Analysis of standard clinical laboratory parameters was performed at the Institute of Clinical Chemistry and Laboratory Diagnostics at the University Hospital Jena and included cholesterol, triglycerides, C-reactive protein (CRP), glomerular filtrating rate (GFR), creatinine, brain natriuretic peptide (BNP) and alanine aminotransaminase (ALAT). Total cholesterol was routinely measured according to manufacturer´s instructions (Cholesterol Assay on ARCHITECT c systems, Abbott Laboratories, USA).

Metabolite concentrations of six analytical classes (40 acylcarnitines, 21 amino acids, 21 biogenic amines, 90 glycerophospholipids, 15 sphingolipids and 1 sugar) were measured in EDTA plasma according to the manufacturer´s protocol (AbsoluteIDQ™ kit p180 [Biocrates Life Science AG, Innsbruck, Austria]). Mass spectrometric analysis was performed on an API4000 liquid chromatography tandem mass spectrometry (LC–MS/MS) system (AB Sciex, Framingam, MA, USA) equipped with an electrospray ionization source, a CTC PAL autosampler (CTC Analytics AG, Switzerland), and the Analyst 1.6.2 software (AB Sciex). Evaluation of calibration curves, quality controls and samples was performed in the MetIQ software package, which is an integral part of the AbsoluteIDQ™ kit. Data were normalized to a reference sample (mean of threefold measurement) on the same plate. Concentration data were exported for the following statistical analysis. Some metabolites, especially glycerophospholipids, were determined only semi-quantitatively, using non-physiological or similar standards.

### Statistical analysis

Statistical analysis was performed using SPSS (RRID:SCR_002865, version 25.0, IBM, USA). Data are expressed as means ± standard deviation (SD) and were compared by student’s *t* test. Comparison of metabolite measurements between pre-TAVR and post-TAVR was performed using the paired *t* test. The correlation between age and metabolites (HC versus pre-TAVR and HC versus post-TAVR) was assessed by linear regression. Adjustment for multiple testing was performed using the Bonferroni correction. Pearson’s correlation coefficient (*r*) was used to investigate the correlation between two variables. An *r* value > 0.5/− 0.5 and a *p* value ≤ 0.05 were considered to be statistically significant.

Heatmaps and principal component analysis (PCA) scores plots were created with the free available MetaboAnalyst (RRID:SCR_015539) 4.0 software [29, 30]. Data were normalized for each metabolite (autoscaling method, mean-centered and divided by the standard deviation of each variable). A hierarchical clustering in form of a dendrogram for the metabolites using the Pearson distance and the average algorithm was performed.

## Results

The demographic and laboratory data of the patient groups are shown in Table 1. There were no differences in sex and BMI, but patients diagnosed with HGAS were significantly older than HC (78.8 ± 7.01 vs. 66.3 ± 6.72) and suffered more often (but not significantly) from diabetes mellitus. Left ventricular ejection fraction and end-diastolic diameter were within the physiological range in both groups. Laboratory values for triglycerides, creatinine and ALAT revealed no relevant differences between the groups, whereas cholesterol, GFR and CRP showed significant alterations between HC and the TAVR groups, but not between pre-TAVR and post-TAVR. BNP was significantly different between the HGAS patients pre- and post-TAVR as well as compared to HC (control 46 ± 35 pg/ml vs. pre-TAVR 607 ± 868 pg/ml vs. post-TAVR 221 ± 348 pg/ml).

**Table 1:** Basic and laboratory characteristics of the study population

	HC (*n* = 20)	pre-TAVR (*n* = 30)	post-TAVR (*n* = 30)	*p* value
Age (years)	66.3 ± 6.7	78.8 ± 7.1	–	< 0.001
Sex (m/f [%])	65/35	50/50	–	0.29
BMI (kg/m^2^)	26.0 ± 4.1	28.3 ± 5.3	–	0.107
Diab. mell. (%)	10	23.3	–	0.119
LVEF (%)	61.9 ± 6.0	56.7 ± 14.4	58.6 ± 11.4	0.307
LVEDD (mm)	42.7 ± 6.7	48.7 ± 7.2*	46.4 ± 7.9	0.012
Cholesterol (mmol/l)	5.92 ± 0.92	4.88 ± 1.63*	4.48 ± 1.05^#^	< 0.001
Triglycerides (mmol/l)	1.46 ± 0.57	1.64 ± 0.81	2.09 ± 2.67	< 0.402
Creatinine (µmol/l)	72.4 ± 10.6	105.0 ± 65.3*	112.3 ± 84.1	0.034
CRP (mmol/l)	2.27 ± 0.61	9.05 ± 14.91*	4.79 ± 5.25^#^	0.048
GFR (ml/min)	82.4 ± 13.7	61.8 ± 20.7*	58.4 ± 19.9^#^	< 0.001
BNP (pg/ml)	46 ± 35	607 ± 868*	221 ± 348^§^	0.003
ALAT (µmol/l)	0.40 ± 0.13	0.37 ± 0.16	0.33 ± 0.19	0.097

*HC* healthy controls, *TAVR* transcatheter aortic valve replacement, *BMI* body mass index, *LVEF* left ventricular ejection fraction, *LVEDD* left ventricular end-diastolic diameter, *CRP* C-reactive protein, *GFR* glomerular filtrating rate, *BNP* brain natriuretic peptide, *ALAT* alanine aminotransaminase. *p* < 0.05 is considered to be statistically significant by *t* test

^*^p < 0.05 between HC and pre-TAVR

^#^*p* < 0.05 between HC and post-TAVR

^§^*p* < 0.05 between pre-TAVR and post-TAVR

The comparison of echo parameters between pre- and post-TAVR revealed significant differences in IVSD, LV mass and LVMI, whereas LVDS, LV shape (concentric vs. eccentric) and pacemaker dependency showed no alterations (see Table 2).

**Table 2:** Echo parameter of the study population pre- and post-TAVR

	pre-TAVR (*n* = 30)	post-TAVR (*n* = 30)	*p* value
LVEF (%)	56.7 ± 14.4	58.6 ± 11.4	0.307
LVEDD (mm)	48.7 ± 7.2	46.4 ± 7.9	0.278
LVDS (mm)	32.2 ± 8.3	31.1 ± 8.4	0.655
IVSD (mm)	15.5 ± 2.7	13.3 ± 2.5	0.002*
LVPWD (mm)	14.6 ± 2.9	13.4 ± 2.8	0.138
LV mass (g)	312.9 ± 94.9	249.0 ± 93.9	0.023*
LVMI (g/m^2^)	167.6 ± 50.3	130.0 ± 39.1	0.004*
LVS (conc [%])	88.9	85.2	0.692
PD (%)	16.6	23.3	0.527

*TAVR* transcatheter aortic valve replacement, *LVEF* left ventricular ejection fraction, *LVEDD* left ventricular end-diastolic diameter, *LVDS* left ventricular end-systolic dimension, *IVSD* interventricular septal thickness at end-diastole, *LVPWD* left ventricular posterior wall thickness at end-diastole, *LVMI* left ventricular mass index, *LVS* left ventricular shape (concentric vs. eccentric), *PD* pacemaker dependency; *p < 0.05 is considered to be statistically significant by *t* test

### Comparison of HGAS pre-TAVR versus HC shows significant alterations in metabolites

**Fig. 1:**
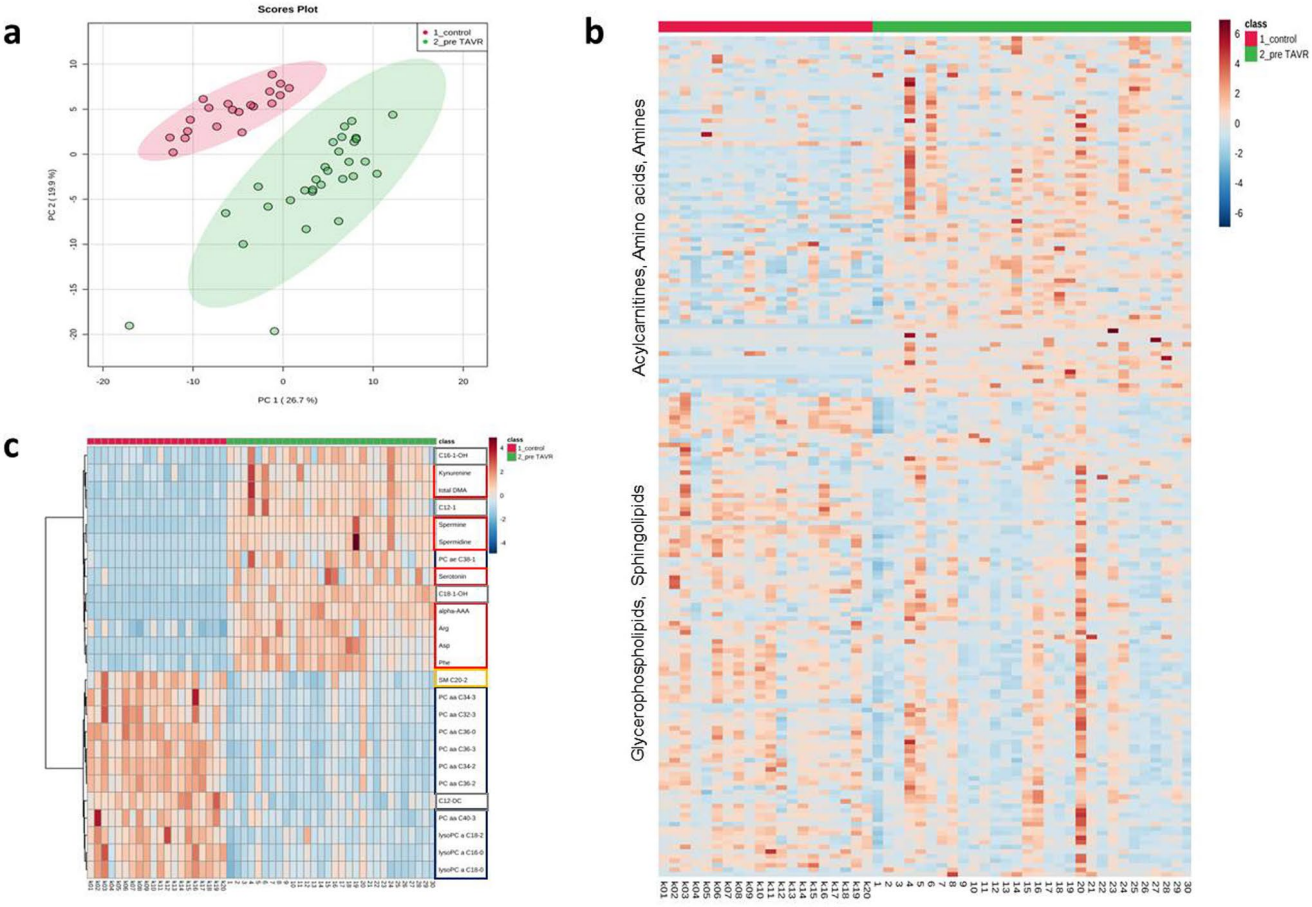
Comparison of metabolite concentrations between patients with aortic stenosis (pre-TAVR) and healthy controls shows significant differences. **a** Principal component analysis of control (red) vs. pre-TAVR (green) showing clear differences between the groups without remarkable outliers. **b** Hierarchical cluster analysis (heatmap) showing increased levels of most ACs, amino acids and biogenic amines and decreased levels of most glycerophospholipids and SMs in the pre-TAVR group compared to healthy controls. Colors indicate metabolite concentration (see color scale bar). Columns represent the samples and rows the metabolites. **c** Heatmap showing differences of selected top 25 metabolites between the pre-TAVR and control group combined with a 2D hierarchical cluster analysis. The dendrogram for the metabolites on the left use the Pearson distance and the average algorithm for clustering. Right side: ACs (green), amino acids and biogenic amines (red), PCs (blue), SM (yellow). TAVR transcatheter aortic valve replacement

To obtain information on differences in the metabolome, we first compared plasma levels from HGAS patients before TAVR with controls. Presented in form of a PCA (Fig. 1a) and a heatmap (Fig. 1b), the graphic panels give an overview of the significant changes in metabolites. Of the 188 measured metabolites, 26.6% (*n* = 50) were significantly altered in patients with HGAS pre-TAVR. In detail, in HGAS pre-TAVR, 5 out of 40 (4 increased, 1 decreased) acylcarnitines (ACs) as well as 10 out of 42 (all increased) amino acids and biogenic amines were significantly altered when compared to HC. Numbers of evaluated glycerophospholipids (25/90 decreased, 4/90 increased) and sphingomyelins (6/15 decreased) were significantly reduced in the HGAS pre-TAVR group compared to HC. We also found a higher sphingomyelin:phosphatidylcholine (SM:PC) ratio (5.5:1 in AS vs. 6.5:1 in HC, *p* = 0.002) as well as a lower lysoPC:PC ratio (12:1 in AS vs. 10:1 in HC, *p* = 0.018) in AS plasma compared with HC. The most altered metabolite levels are shown in the top 25 heatmap (Fig. 1c). Though all classes of measured metabolites are represented, there is a clear drift to reduced long-chain phosphatidylcholines and increased biogenic amines and amino acids.

### Comparison of HGAS pre-TAVR versus post-TAVR reveals reversible changes

**Fig. 2:**
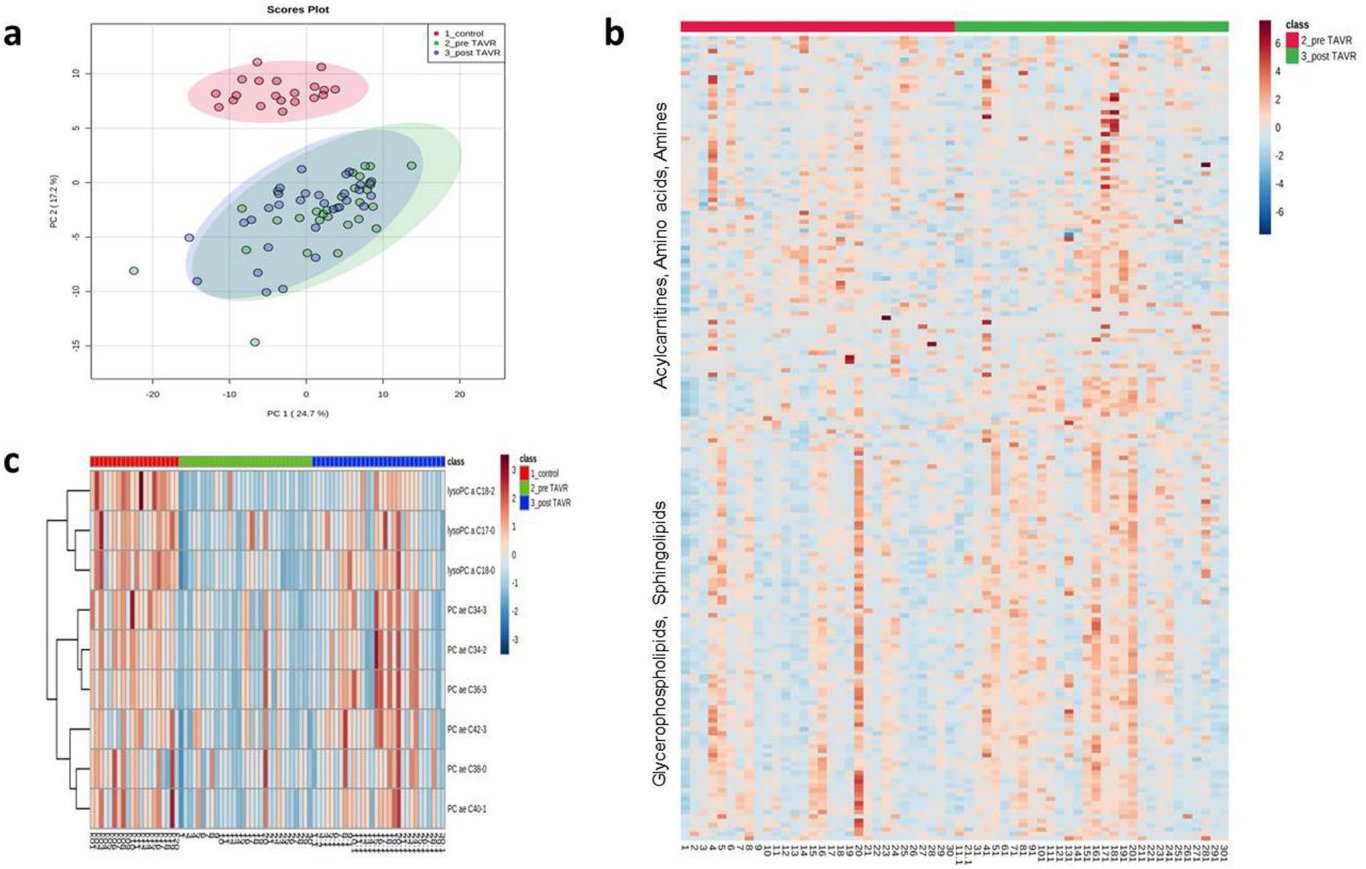
Comparison of metabolite concentrations in patients with aortic stenosis pre-TAVR vs. post-TAVR vs. healthy controls results in several reversible metabolites. **a** Principal component analysis of the three groups (controls: red, pre-TAVR: green, post-TAVR: blue) showing only clear differences in comparison to the control group. **b** Heatmap showing general metabolomic profile of the pre-TAVR and post-TAVR group. **c** Heatmap of 9 selected metabolites showing the reversible differences in metabolite concentrations in the post-TAVR group compared to pre-TAVR and healthy controls. Hierarchical cluster analysis on the left use the Pearson distance and the average algorithm for clustering. TAVR transcatheter aortic valve replacement

**Fig. 3:**
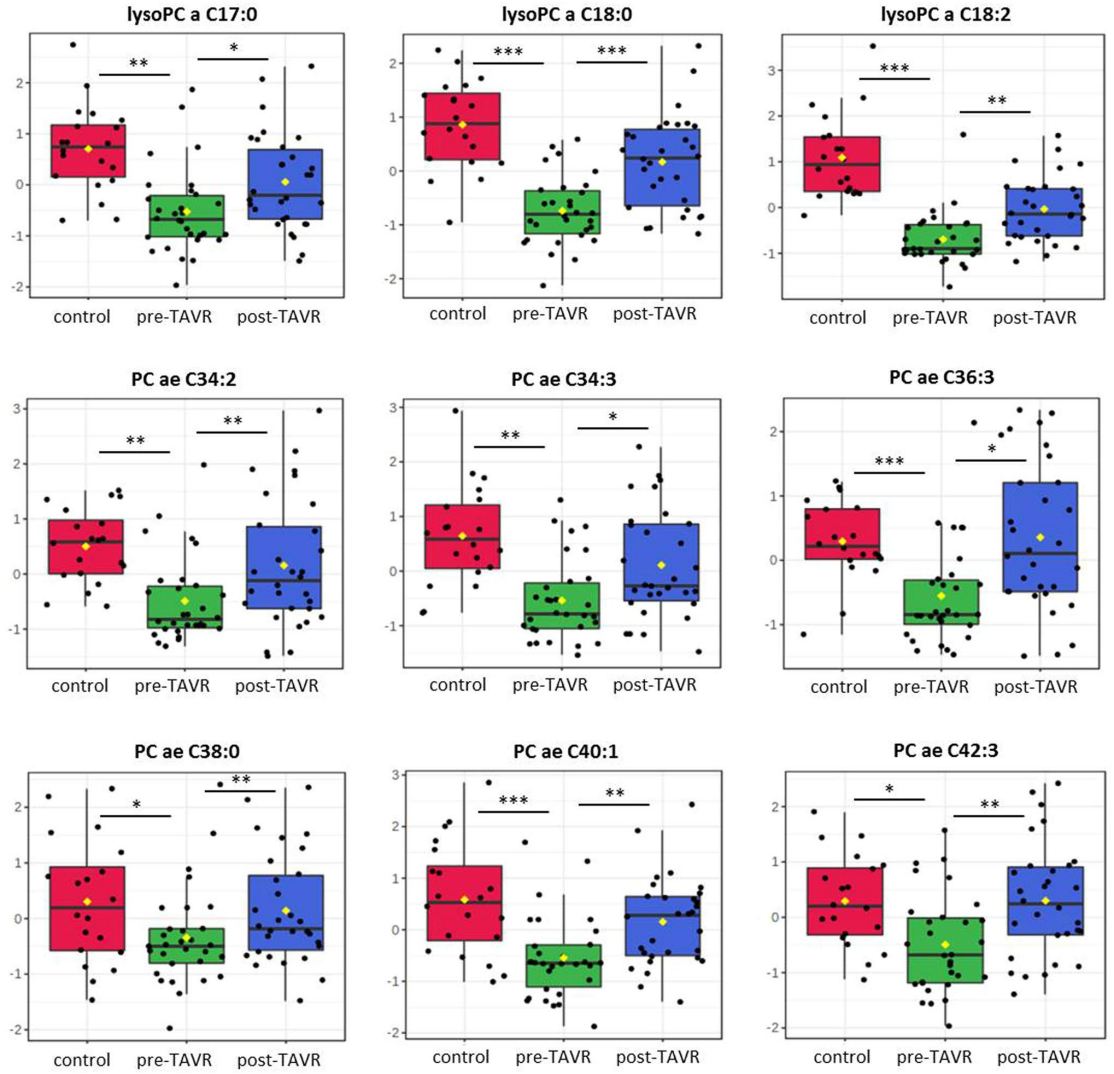
Quantitative comparison of significantly altered metabolites in the post-TAVR group compared to pre-TAVR and controls. Box plots including individual data points of three lysoPCs and six PCs. Data presented as medians ± SE. *p < 0.05, **p < 0.01 and ***p < 0.001 when compared by two-sided *t* test. TAVR transcatheter aortic valve replacement

To show possible differences as the consequence of surgery treatment, we measured in the second step the metabolite levels of HGAS patients before (pre-TAVR group) as well as 6 weeks after the replacement (post-TAVR group). In contrast to HC, PCA and heatmap show rare discrimination between the pre- and the post-TAVR groups (Fig. 2a, b). Here, only 10.1% (*n* = 19) of the 188 metabolites were altered significantly. In detail, 2/42 (all increased) amino acids and biogenic amines and 17/90 glycerophospholipids (all increased) displayed differences. In a subsequent investigation, we found 9 metabolites altered between pre-TAVR and HC as well as between pre-TAVR and post-TAVR, but not between post-TAVR and HC (Fig. 2c). Quantitative analysis represents three lysoPCs (lysoPCaC17:0, lysoPCaC18:0 and lysoPCaC18:2) and several long-chain PCs (PCaeC34:2, PCaeC34:3, PCaeC36:3, PCaeC38:0, PCaeC40:1 and PCaeC43:3) to show reversible concentration levels six weeks after TAVR treatment (Fig. 3). Furthermore, the post-TAVR SM:PC ratio as well as the lysoPC:PC ratio showed no longer a significant difference compared to HC, respectively.

### Correlation of metabolites with clinical parameters

**Fig. 4:**
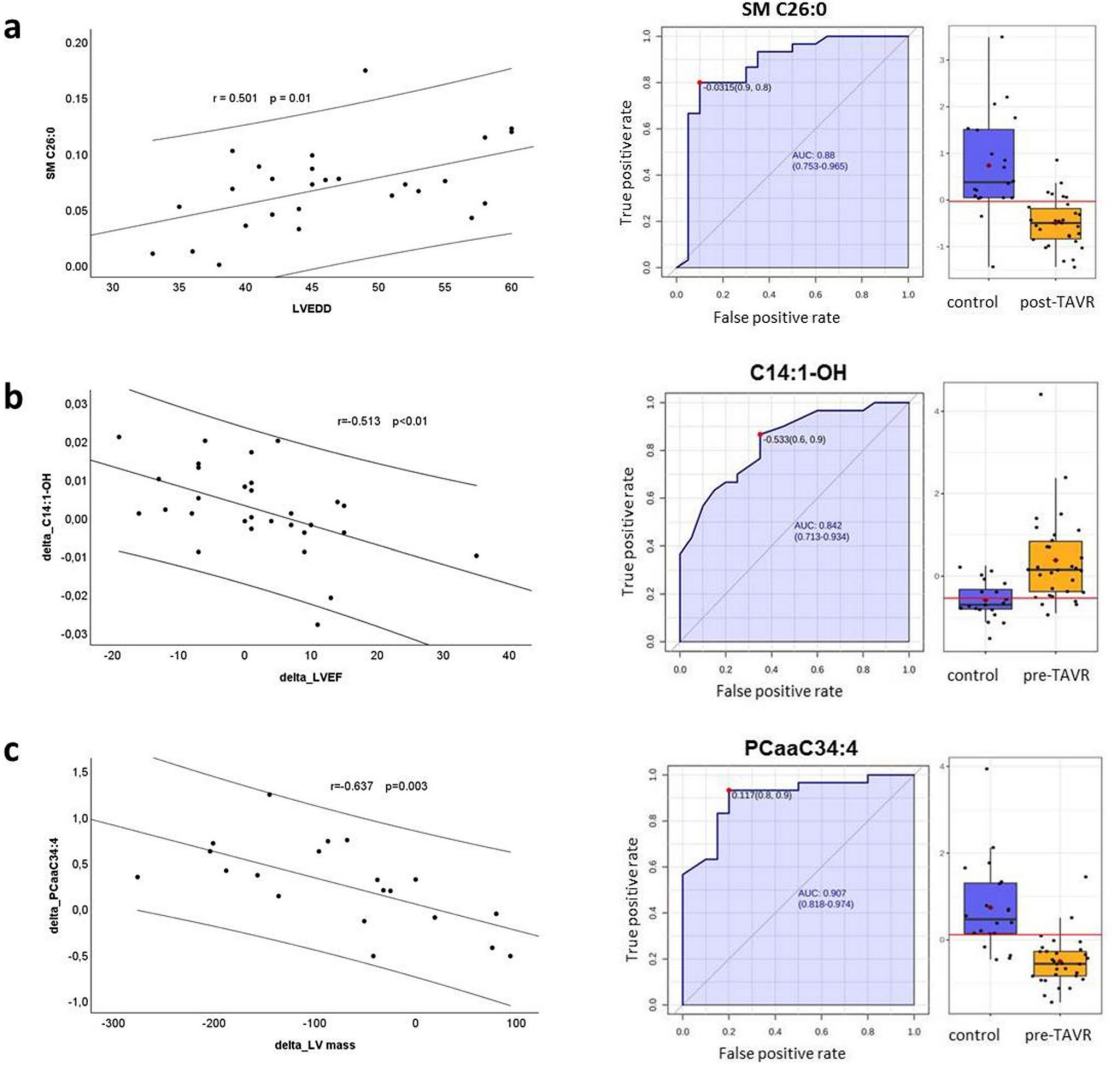
Correlation between significantly altered metabolites and clinical parameters of the study population. Pearson correlation, Receiver operator characteristic curve (ROC) and box plot of selected metabolite comparing **a** LVEDD and the sphingomyelin SM C26:0 between HC and post-TAVR, **b** δ LVEF and the acylcarnitine C14:1-OH between pre- and post-TAVR and **c** δ LV mass and the phosphatidylcholine PCaaC34:4 between pre- and post-TAVR. Correlation graphs including regression line and 95% confidence interval. *LVEDD* left ventricular end-diastolic diameter; *LVEF* left ventricular ejection fraction; *r* Pearson correlation coefficient; *p* probability value

To identify potential biomarkers for the evaluation of the clinical course, we performed a correlation analysis between significant altered metabolites and the clinical parameters presented in Tables 1 and 2. In the pre-TAVR group, there was a strong correlation of several ACs with creatinine, GFR (*r* = − 0.725) and BNP (*r* = 0.664); several glycerophospholipids with cholesterol, creatinine and BNP; few biogenic amines with creatinine, GFR and BNP and several SMs with cholesterol (*r* = 0.847, *p* < 0.01) (see Online Resource 1). 6 weeks after TAVR, we found again strong correlations between few ACs and creatinine, GFR and BNP; few amino acids as well as biogenic amines and LVEF, triglycerides, creatinine, GFR and BNP; several glycerophospholipids and cholesterol, creatinine, CRP, BNP and LVEDD and several SMs and cholesterol and LVEDD. Figure 4a shows exemplarily the correlation and ROC analysis of sphingomyelin SM C26:0 (*r* = 0.501, *p* = 0.01, AUC = 0.88) when comparing the post-TAVR group with HC. Additionally, we found several metabolites to have strong correlations both pre-TAVR and post-TAVR: ACs with creatinine (*n* = 2), GFR (*n* = 3) and BNP (*n* = 1); biogenic amines with GFR (*n* = 2) and BNP (*n* = 1); PCs with cholesterol (*n* = 10) and BNP (*n* = 1) and SMs with cholesterol (*n* = 4). Among these, one PC did not only show strong correlation with cholesterol pre- and post-TAVR, but were also reversible in their concentration (lysoPCaC18:0, *r* = 0.544 and 0.529, respectively). Besides, a cluster of seven ACs and one biogenic amine pre-TAVR as well as two ACs post-TAVR showed several clinical correlations, revealing the AC C5-M-DC the only metabolite present in both groups (see Online Resource 2).

Correlation statistics between the metabolites and the follow-up parameters revealed significant correlations between the acylcarnitine C14:1-OH and delta (*δ*) LVEF (see Fig. 4b) and alanine and *δ* LVEDD. Besides, three PCs simultaneously correlated with *δ* LV mass, *δ* LVMI and *δ* LVPWD, which indicates a correlation with an improvement of these functional parameters and, therefore, the ability to predict a good outcome after TAVR (see Fig. 4c). Additionally, several PCs and SMs correlated with δ cholesterol, few ACs and AAs/BAs with δ triglycerides, δ creatinine, δ CRP and δ BNP and several AAs with δ ALAT (see Online Resource 1).

## Discussion

The development of high-throughput metabolomic technologies has allowed to investigate large patient cohorts of different disease states and to achieve new inputs for putative diagnostic, prognostic or therapy surveillance markers. Thus, independent studies previously reported metabolic alterations in the failing heart [25, 31, 32].

Aortic stenosis belongs to the most frequent clinical causes of hospitalization and mortality in elderly people and TAVR, being the preferred option for elderly patients, is a well-established interventional therapy for permanent improvement.

We here screened patients with severe AS immediately before and 6 weeks after TAVR to elucidate the individual metabolic state. Compared to controls, we found a distinct metabolic profile in AS patients’ pre-TAVR. We demonstrated that patients with AS have significantly elevated levels of short-chain and long-chain ACs and, during a 6-week follow-up, these levels slightly decrease. ACs are carnitine-bound fatty acyls required for transport of fatty acids into mitochondria. Thus, they play a key role in providing substrates derived from fatty acid uptake to cardiomyocytes [33]. Increased AC plasma levels indicate an incomplete fatty acid oxidation and deficiencies in mitochondrial AC uptake [34]. In heart failure patients, elevation of ACs is associated with poor long-term outcome [35]. Furthermore, the effects of long-chain ACs are pro-inflammatory and arrhythmogenic [36, 37]. In a previous study in patients undergoing TAVR, a metabolomics study revealed the putative prognostic marker 5-adenosylhomocysteine for the prediction of acute kidney injury [27]. Our results are in accordance with these findings, since we could show a strong correlation between ACs and GFR as well as with creatinine. Of importance, we found the AC C5-M-DC to have strong correlations with BNP, GFR and creatinine both pre- and post-TAVR. C5-M-DC or methylglutaryl-l-carnitine has a biological role as membrane stabilizer and energy source and is described as a diagnostic metabolite of 3-hydroxy-3-methylglutaryl-coenzyme A lyase deficiency [38]. Furthermore, the AC C14:1-OH (or hydroxytetradecenoylcarnitine) was found to show correlation with the improvement of LVEF. C14:1-OH has already been described as positively correlated with carotid plague area in an aging-related disease study [39]. Together, our results show elevated ACs as a consequence of incomplete fatty acid oxidation in mitochondria, a process which is only partially reversible six weeks after treatment. More important are plasma levels of the short-chain ACs C5-M-DC and C14:1-OH, which show diagnostic potential even on short-term outcome.

A further analytical class we found to be mainly increased in AS are amino acids and biogenic amines. Enhanced amino acids count for a hypercatabolic state and have been reported in chronic heart failure [40, 41]. Elevated amino acids as well as creatinine are postulated to be side effects of the renal impairment commonly observed in heart failure [42]. One of the most significantly altered metabolites in this group is kynurenine, a metabolite of the L-tryptophan pathway. Kynurenine has been recently reported to be significantly elevated in patients with coronary artery disease [43] and was suggested to predict cardiovascular prognosis [44]. We demonstrate a strong correlation between kynurenine and GFR and creatinine pre-TAVR as well as between kynurenine and GFR and BNP post-TAVR and, therefore, assume that kynurenine levels are a good indicator not only in AS but also in cardiovascular diseases.

We also demonstrate a significant reduction of phosphatidylcholines (PCs) and sphingomyelins (SMs) in AS. PCs are a major component of biological membranes and play a key role in membrane-mediated cell signaling. They underlie a cyclical synthesis and degradation, resulting in the synthesis of lysophosphatidylcholines (lysoPC). Thus, a possible explanation of the downregulation of PCs is their hydrolysis by phospholipase A2, resulting in one fatty acid and lysoPC, which can be further hydrolyzed by lysophospholipase to free fatty acids. A further reason could be the modification of side chains by oxidation occurring during oxidative stress and inflammation [45].

Decreased plasma PC concentrations have been already found in other disease states like sepsis [46] or pneumonia [47]. Furthermore, lysoPCs are increasingly considered as factors positively associated with cardiovascular and neuronal diseases. However, recent clinical findings of plasma lysoPCs in cardiovascular diseases are controversial. While increased levels in the circulation are associated with the development of atherosclerotic plaques [48], several lipidomic profiling studies showed a negative correlation with cardiovascular diseases [49, 50]. In accordance with these findings [49–51], we can show reduced lysoPC levels in AS and, with the reversal of the decreased lysoPC:PC ratio a clear trend to the normalization of these levels even six weeks after TAVR.

The involvement of lysoPCs in inflammatory processes is mediated, amongst others, by the enzyme NOS3 and NO synthesis and production of antioxidant enzymes [52]. As a consequence, lower lysoPC levels may increase oxidative stress and promote inflammation. In this context, we found the amino acid arginine, which is metabolized to NO, significantly increased in AS compared to HC and, together with the low lysoPC levels, this could be a possible link to inflammatory signaling in AS. The reversibility of the lysoPCs aC17:0, aC18:0 and aC18:2 seems to be a good indicator for further investigations regarding a prognostic marker in AS. Interestingly, the lysoPCs aC17:0 and aC18:2 were selected as biomarkers in myocardial infarction [51]. Of further interest, in the post-TAVR group, we did not find levels of lysoPC comparable to HC but a significant increase in PC:lysoPC between pre-TAVR and post-TAVR. This suggests a normalization of PC:lysoPC levels towards convalescence. Moreover, the structural myocardial changes within 6 weeks represent an ongoing reverse remodeling and the correlation of some PCs with LV mass and LVMI could, therefore, predict a better clinical course.

It is also remarkable that all of the reversible metabolites in this study belong to the class of glycerophospholipids. It seems that PCs and lysoPCs are the analytical classes with the fastest normalization post-TAVR and the fact that several glycerophospholipids are reversible even as early as six weeks after TAVR shows their importance and potential for early prognostic evaluations.

Sphingomyelins (SMs) are also found in membranes and lipoproteins. Circulating plasma levels are discussed to be associated with increased risk of cardiovascular disease. While some studies showed an association of higher SM levels with atherosclerosis or acute myocardial infarction [53, 54], another study demonstrated that high SM levels were not associated with coronary heart disease [55]. In contrast to our results, Doppler et al*.* found increased levels of SMs in aortic tissue in patients with bicuspid aortic valve disease [56]. However, it is comprehensible that tissue levels do not mirror plasma levels of one compound. Because ceramides are produced in part by hydrolysis of SM, the presence of accumulated ceramides in the failing heart is very likely due to SM degradation [57]. Thus, reduced SM levels in our study would further support this assumption. The correlation of some of the investigated SMs with cholesterol pre-TAVR as well as post-TAVR and the correlation of SM C26:0 with LVEDD could be of further interest as prospective markers.

Cardiac interstitial fibrosis is an important key step of cardiovascular remodeling. A recent study assessing the extent of fibrosis in patients suffering from AS and undergoing TAVR could clearly point out its prognostic impact [58]. Unfortunately, in our study, we could not assess fibrosis since we did not performed MRI studies or cardiac biopsies in our patients. Nevertheless, the observed metabolic alterations might be closely linked to the extent of ventricular mass as well as LV mass index, 6 weeks after the procedure. This phenomenon might be due to a reverse remodeling associated with a decrease of cardiac fibrosis. When planning for further studies, it would be of great interest to correlate individual tissue fibrosis levels with metabolomics, both, prior to as well as in the follow-up after TAVR.

Our study has several limitations. First, the case number enrolled in this study is relatively small. As a consequence, the identified metabolites need to be further verified by a larger patient cohort. However, the high ROC AUC values suggest great potential of the selected metabolites even for this small cohort. Furthermore, there was a significant difference in age between the control and the AS group. Therefore, the metabolites were adjusted in a regression model and so the different metabolite levels we found cannot be attributed to age. Though we have a 6-week follow-up of the AS patients and can show first reversible metabolic effects, we have not yet performed follow-up studies over longer time periods (e.g. six month or 1 year) to assess the dynamics of potential biomarkers in progress of the clinical course. Further studies with additional follow-up might, therefore, be of great interest.

To our knowledge, this is the first study investigating the metabolomic profile of patients with AS pre- and six-week post-TAVR compared to healthy controls. The metabolomic approach identified a unique metabolomic fingerprint in patients with AS. We found a panel of metabolites which are suited to distinguish patients with AS from healthy controls, in particular long-chain ACs and long-chain PCs and some biogenic amines. 6 weeks after TAVR, the metabolomic profile is only merely changed, but of importance is the early reversibility of several metabolites, showing potential as early prognostic markers in high-risk patients. Several metabolites show correlation with improved LVEF and LVEDD function or reduced LV mass and, therefore, are suited to predict a better clinical outcome after TAVR. Though metabolites found in plasma cannot mirror pathogenesis, these distinct metabolites can represent a novel class of diagnostic biomarkers connecting the metabolome to transcriptomic and proteomic studies. Further analysis will focus on the identification of the prognostic power of these metabolites and their specific role in the reverse cardiac and vascular remodeling processes in patients with AS following TAVR.

## Electronic supplementary material

Below is the link to the electronic supplementary material.

Supplementary file1 (DOCX 25 kb)

Supplementary file2 (DOCX 25 kb)

Supplementary file3 (XLSX 244 kb)
