# Supplementary material for: Metabolomic profiling of patients with high gradient aortic stenosis undergoing transcatheter aortic valve replacement
Source: Clin Res Cardiol. 2020 Oct 14;110(3):399–410. doi: 10.1007/s00392-020-01754-2 (PMC7907030; doi:10.1007/s00392-020-01754-2)
Supplement: Supplementary file 1 — Supplementary file1 (DOCX 25 kb) [file 392_2020_1754_MOESM1_ESM.docx]

**Metabolomic profiling of patients with high gradient aortic stenosis undergoing transcatheter aortic valve replacement**

**Clinical Research in Cardiology**

Daniela Haase, PhD^1§^, Laura Bäz, MD^1§^, Tarek Bekfani, MD^1^, Sophie Neugebauer, PhD^2^, Michael Kiehntopf, MD, PhD^2^, Sven Möbius-Winkler, MD^1^, Marcus Franz, MD^1#^, P. Christian Schulze, MD^1#^

^1^ Department of Internal Medicine I, Division of Cardiology, Angiology, Pneumology and Intensive Medical Care, University Hospital Jena, Friedrich-Schiller-University, Jena, Germany

^2^ Department of Clinical Chemistry and Laboratory Diagnostics, University Hospital Jena, Friedrich-Schiller-University, Jena, Germany

^§^,^#^ Equally contributing authors

**Corresponding author**

P. Christian Schulze, MD, PhD

E-Mail: [christian.schulze@med.uni-jena.de](mailto:christian.schulze@med.uni-jena.de)

**Online Resource 1: Number of correlations between significant altered metabolites and clinically important parameters**

acylcarnithines amino acids, biogenic phosphatidylcholines sphingomyelines

(n=40) amines (n=42) (n=90) (n=15)

pre / post pre / post pre / post pre / post

LVEF 0 / 0 0 / 1 0 / 0 0 / 0

LVEDD 0 / 0 0 / 0 0 / 1 0 / 1

LVDS 2 / 0 1 / 0 0 / 0 0 / 0

IVSD 0 / 1 0 / 0 0 / 0 0 / 0

LVPWD 0 / 0 0 / 0 1 / 0 0 / 0

LV mass 1 / 0 0 / 0 0 / 0 0 / 0

LVMI 1 / 0 0 / 0 0 / 1 0 / 0

Cholesterol 0 / 0 0 / 0 31 / 27 8 / 7

Triglycerides 0 / 0 0 / 1 0 / 0 0 / 0

Creatinine 15 / 5 3 / 3 2 / 4 0 / 0

CRP 0 / 0 0 / 0 0 / 2 0 / 0

GFR 12 / 3 3 / 3 0 / 0 0 / 0

BNP 11 / 3 1 / 4 3 / 4 0 / 0

ALAT 0 / 0 0 / 0 0 / 0 0 / 0

δ LVEF 1 0 0 0

δ LVEDD 0 1 0 0

δ LVDS 1 4 3 0

δ IVSD 1 0 0 0

δ LVPWD 1 0 11 0

δ LV mass 1 2 6 0

δ LVMI 0 2 5 0

δ Cholesterol 0 0 3 10

δ Triglycerides 2 2 1 0

δ Creatinine 2 1 1 0

δ CRP 1 1 1 0

δ GFR 0 0 0 0

δ BNP 4 0 1 0

δ ALAT 0 5 1 0

LVEF left ventricular ejection fraction, LVEDD left ventricular end-diastolic diameter, LVDS left ventricular end-systolic dimension, IVSD interventricular septal thickness at end-diastole, LVPWD left ventricular posterior wall thickness at end-diastole, LVMI left ventricular mass index, CRP C-reactive protein, GFR glomerular filtrating rate, BNP brain natriuretic peptide, ALAT alanine aminotransaminase. δ difference between post-TAVR and pre-TAVR.
