# Supplementary material for: Metabolomic profiling of patients with high gradient aortic stenosis undergoing transcatheter aortic valve replacement
Source: Clin Res Cardiol. 2020 Oct 14;110(3):399–410. doi: 10.1007/s00392-020-01754-2 (PMC7907030; doi:10.1007/s00392-020-01754-2)
Supplement: Supplementary file 2 — Supplementary file2 (DOCX 25 kb) [file 392_2020_1754_MOESM2_ESM.docx]

**Metabolomic profiling of patients with high gradient aortic stenosis undergoing transcatheter aortic valve replacement**

**Clinical Research in Cardiology**

Daniela Haase, PhD^1§^, Laura Bäz, MD^1§^, Tarek Bekfani, MD^1^, Sophie Neugebauer, PhD^2^, Michael Kiehntopf, MD, PhD^2^, Sven Möbius-Winkler, MD^1^, Marcus Franz, MD^1#^, P. Christian Schulze, MD^1#^

^1^ Department of Internal Medicine I, Division of Cardiology, Angiology, Pneumology and Intensive Medical Care, University Hospital Jena, Friedrich-Schiller-University, Jena, Germany

^2^ Department of Clinical Chemistry and Laboratory Diagnostics, University Hospital Jena, Friedrich-Schiller-University, Jena, Germany

^§^,^#^ Equally contributing authors

**Corresponding author**

P. Christian Schulze, MD, PhD

E-Mail: [christian.schulze@med.uni-jena.de](mailto:christian.schulze@med.uni-jena.de)

**Online Resource 2: Summary of metabolites with several clinical correlations**

Metabolite (pre-TAVR) r (BNP) r (GFR) r (creatinine)

C5-DC (C6-OH) 0.564 -0.631 0.827

**C5-M-DC** 0.651 -0.650 0.883

C6 (C4:1-DC) 0.507 -0.621 0.727

C14:1 0.599 -0.554 0.705

C14:1-OH 0.620 -0596 0.730

C14:2 0.664 -0.583 0.713

C18:1 0.631 -0.581 0.586

total DMA 0.645 -0.747 0.822

Metabolite (post-TAVR)

**C5-M-DC** 0.754 -0.603 0.599

C5-OH (C3-DC-M) 0.630 -0.689 0.864

Metabolite (post-pre) r (δ LV mass) r (δ LVMI) r (δ LVPWD)

PCaaC34:4 -0.650 -0.628 -0.576

PCaeC34:0 -0.522 -0.524 -0.765

PCaeC38:1 -0.517 -0.552 -0.508

r Pearson correlation coefficient; acylcarnitine C5-M-DC present in two groups (bold)
